# Research on soybean curd coagulated by *lactic acid bacteria*

**DOI:** 10.1186/2193-1801-2-250

**Published:** 2013-05-31

**Authors:** Wang Jianming, Lin Qiuqian, Wang Yiyun, Chen Xi

**Affiliations:** Key laboratory of Food Nutrition and Safety, Ministry of Education, College of Food Engineering and Biotechnology, Tianjin University of Science & Technology, No.29, at the 13th Avenue, Tianjin Economical-Technological Development Area, Tianjin, Tanggu District, 300457 P. R. China

**Keywords:** *Lactic acid bacteria*, Soybean curd coagulation, Gelation

## Abstract

Chinese traditional soybean curd is coagulated by calcium salt. In order to investigate the feasibility of soybean curd coagulation by *lactic acid bacteria*, we studied the effective factors in soybean curd coagulation when using common soybean curd strains. In soybean curd concentration, incubating time and temperature as well as the inoculated amount and the edible gum additives were studied as the coagulating factors of soybean curd fermented by lactic acid bacteria. Based on the single factor and orthogonal experiment design, the optimized conditions of lactic acid bacteria fermentation curd was determined as follows: soybean curd concentration 12.5%(v/v)and the fermentation conducted at 42°C for 5 hours when the inoculating amount was 4.0% and edible gum additives was 1.4% (carrageenan 1.0% (m/v), soluble starch 0.4% (m/v)). Under the optimum conditions, the water-holding rate of the bean curd was measured as 69.82%, and the gel strength was 25.6 g/cm^2^. Compared with the traditional tofu coagulated by calcium salt, our products has less off-flavor and softer texture, which was accepted as new type of soybean curd according to the overall sensory evaluation

## Introduction

Soybean curd, one kind of Chinese traditional food, contains high quality protein that can be easily digested and no cholesterol (Lei et al. 2007; Lijun Guan^2009^). The incorporation of soybean curd into a western diet could be an important means of preventing and treating chronic diseases, such as cancer and cardiovascular diseases as supported by epidemiological studies (Xinghua Guo 2008). The ratio of polyunsaturated to saturated fatty acids of soybean curd is higher than cheese (Zhihong Qiao 2008). Dairy products and meat are the primary contributors to accumulate polychlorinated dibenzo-*p*-dioxins and dibenzofurans (PCDD/F) up to the reproductive age. Hsiu Ling Chen determined consumption of soybean curd was negatively correlated with serum PCDD/F levels (Jiebing Yang 1999).

Coagulation of soybean curd is the most important step in the soybean curd making process. The most widely used coagulant of Chinese traditional soybean curd is salt-coagulant, such as gypsum and bittern. Gypsum soybean curd has good water retentivity, smooth texture and high product yield. But the solubility of gypsum is low, and the speed of solidification slow. In the practical production, it’s difficult to control the correct dosage of gypsum. When adding too much gypsum, soybean fragrance will lose and soybean curd will be bitter. The solidification speed of bittern soybean curd is fast, but protein network structure is easy to shrink. Thus, bittern soybean curd has no good water retentivity and product yield is low.

The coagulant of physalis soybean curd is physalis with lactic acid, which is made from the fermented yellow serofluid under natural conditions. Physalis is rich in lactic acid bacteria, which can break down protein, fat and polysaccharide. Physalis soybean curd is of good water retentivity, fine texture and subtle soybean fragrance. However, the usage amount of physalis depends entirely on worker’s experience, without strict standards, which leads to commercial production failure. Since there are similarity in casein coagulation and the soy protein coagulation with acid, here in this paper, we followed the main procedure of yogurt manufacture and investigated the feasibility of soybean curd coagulation by lactic acid bacteria, in order to innovate the traditional soybean curd coagulation.

### Materials and equipments

### Raw materials

Soybean sold in Desco Super Makert, Tianjin, P.R. China.

### Experimental strains

CYY-122 *Bulgarians* and *Streptococcus thermophilus*; SVV-21 *Bulgarians* and *Streptococcus thermophilus* (All samples from Netherlands DSM Company).

### Reagent

Soluble starch (purity in analysis level; Tianjin North TianYi Chemical Reagent Factory); Sodium alginate (purity in chemical level; Tianjin YongDa Chemical Reagent Development Center); Restore gum sold in market; Xanthan gum (from Golden phenix carrageenan limited liability company); fresh milk (from Tianjin Haihe dairy Co., Ltd.).

### Experimental equipments

AB204-N electronic analytic balance (from Mettler - Toledo equipment Co., Ltd.); 752E UV/Visible spectrophotometer (from Shimadzu Corporation); FE-20 pH meter (from Mettler - Toledo equipment Co., Ltd.); DGG-101 Electric blast-drying oven (from Tianjin Tianyu Mechanic and electric Co., Ltd.); DHP Electric heating constant temperature incubator (from Huangshi, Hubei Medical instrument factory); WYT-4 Portable glucose meter (from Quanzhou Zhongyou Optical instruments Co., Ltd.); W_4_ Colloid mill (from Wenzhou Donghai Machine building plant).

## Results and discussion

### Effects of soy milk concentration (In terms of the content of soluble solids) on soybean curd

Compound the soy milk by the rates of bean and water respectively as 1:6,1:7,1:8,1:9, then test soluble solids content, and select the soy milk whose soluble solids content is 9.5%, 11.0%, 12.5%, 13.0%, respectively, as examples. Each of the samples has been inoculated CYY-12 and SVV-21 as the amount of 1.0% of soy milk’s volume, and put them into the constant temperature incubator to ferment for 5h while other conditions remain the same. After that, keep their temperature and let them to be formed, then measure each sample’s soybean curd yield and do sensory evaluation.

**Figure 1:**
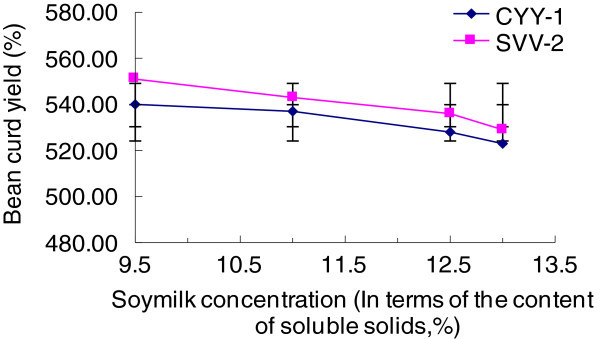
**Effects of soy milk concentration on soymilk curd.**

The effect on soybean curd yield of the soy milk whose soluble solids content is 9.5%, 11.0%, 12.5%, 13.0%, respectively, is shown on the Figure 1.

From Figure 1 we can see that the difference of stains has little influence on soybean curd yield, but the yield decreased as soy milk concentration (the content of soluble solids) increased. Because the lactic acid bacteria produced lactic acid in the fermentation of soy milk, it reduced the pH of the soy milk solution which made the pH approach to the isoelectric point of the protein. That made the protein change its state from sol to gel, and the protein gel network contains water, so the soybean curd yield increased. However, the proportion of water in the soybean curd is much larger than the protein ratio, so as soy milk concentration increased, even though the increase in protein content, the quality of the water molecules is much smaller than the protein’s, thus the increase in the number of protein molecules was much less than the decrease in number of water molecules. As a result, the protein gel network contained less water, or it formed into an incomplete gel network since the lack of water molecules, so that the soybean curd yield decreased comparatively. By the sensory evaluation, the soy milk whose concentration is 12.5% or 13.0% had the fragrance of bean, and their block types were complete with delicate texture. However, considering the cost of production, 12.5% should be the best choice.

### Effects of incubation time on soybean curd

Select the soy milk whose soluble solids content is 12.5% to inoculate CYY-12 and SVV-21 respectively, and culture them under the temperature of 37°C, then observe the curding condition and measure the pH value.

**Figure 2:**
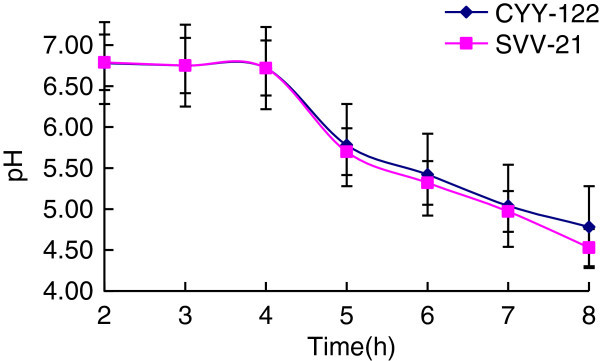
**Influence from pH value on curd procession of two stains.**

After two strains having been cultured for different time, the change of pH value has been shown in Figure 2.

As the extension of fermentation time, fermentation products have further accumulation and acid production increases. Extending the fermentation time will not only increase the fermentation cycle and contaminate other bacteria more easily, but also have an impact on flavor and organizations state. Besides, around the pH value of 9.5, soy milk solidified under the effect of coagulant. As can be seen from the Figure 2, sharper decline in pH value was shown with the fermentation time from 4h to 6h.

Under different culture time, the sensory evaluation of curd was as shown on Table 1. Additionally, we also make a comparison on those sensory indexes with traditional soybean curd under the same cultivated conditions, which can help us judge two kinds of curds processed by new or traditional methods. This result was shown on Table 2.

**Table 1:** **Sensory evaluation of the different culture time**

T/h	The average score			
	Color	Flavor	Morphology	Taste
1	3.0	2.5	1.0	3.5
2	3.0	2.5	2.0	3.5
3	3.0	3.3	3.0	3.5
4	3.3	3.6	4.0	4.0
5	4.5	4.5	4.5	4.5
6	4.5	4.5	4.5	4.0
7	4.6	3.5	4.0	4.0
8	4.7	3.0	4.0	3.0

**Table 2:** **Sensory evaluation of two different curds**

Two kinds of curds	The average score			
	Color	Flavor	Morphology	Taste
Soybean curd processed by lactic acid bacteria	4.6	4.0	4.0	4.5
Traditional soybean curd	4.5	3.5	4.0	3.7

As it is shown in the Table 1, from 2h to 6h, the aroma was getting light while the acidity was increasing. Taking comprehensive consideration of fermentation time, pH value, and the aroma of the product, etc., the best fermentation time is 5h.

From the above description and data base, it is concluded that coagulating by the chosen strains can provide products’ with lovely appearance and flavor, which is much better than the current traditional methods.

### Effects of temperature on soybean curd

Select the soy milk whose soluble solids content is 12.5% to inoculate CYY-12 and SVV-21 respectively, set them under the temperature of 37°C, 39°C, 40°C, 42°C, respectively. Measure the curding time, soybean curd yield and water holdup.

**Figure 3:**
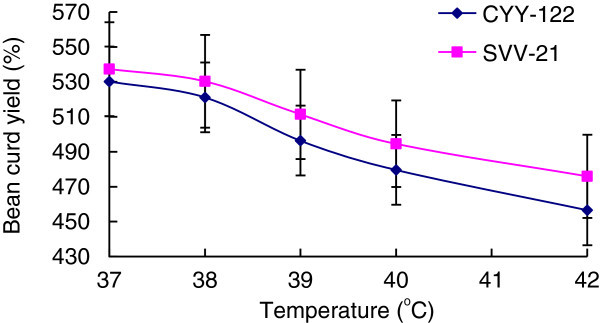
**Effect of temperature on soymilk curd.**

The influence on soybean curd’s water capacity from different temperature as 37°C, 38°C, 39°C, 40°C, 41°C, 42°C has been shown on Figure 3.

**Figure 4:**
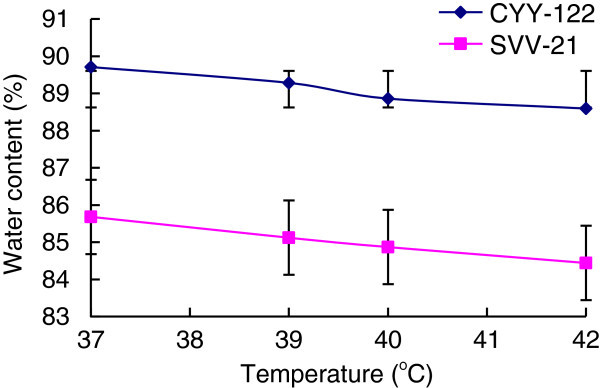
**Effect of different fermentation temperature on soymilk’s water capacity.**

The optimum growth temperature of bacteria used in this experiment is between 37°C and 42°C, therefore choose 37°C ~ 42°C range as a single factor. From Figure 3 and Figure 4 we can see that as the temperature rising, both soybean curd yield and moisture content rate produced by two strains decreased, but at 37°C the soybean curd yield and moisture content rate both reached their maximum, and at these temperature lactic acid bacteria can accumulate the largest amount of metabolism material in fermentation, which is beneficial to soybean curd curding, so 37°C is optimum fermentation temperature of experimental strains.

Since the optimal temperature for our method is easy to reach, we can see that being fermented by strains we chose is generally practical.

### Effects of stains on soybean curd

#### Effects of stains’ kinds on soybean curd

Select the soy milk whose soluble solids content is 12.5% to inoculate CYY-12 and SVV-21 respectively, and culture them under the temperature of 37°C for 5h. Measure each sample’s soybean curd yield and do sensory evaluation.

There are little differences on soybean curd yield between using two stains in fermentation, and no significant differences in the product’s color, flavor, morphology, and taste. Therefore, we can consider mixing two stains in fermentation, or adding the amount of strains to study the impact on soybean curd quality.

### Effects of strains’ adding amount on soybean curd

Select the soy milk whose soluble solids content is 12.5% to inoculate CYY-12 and SVV-21 as the amount of 1.0%, 2.0%, 3.0%, 4.0% of soy milk’s volume, respectively, and culture them under the temperature of 37°C for 5h. Measure each sample’s soybean curd yield and do sensory evaluation.

**Figure 5:**
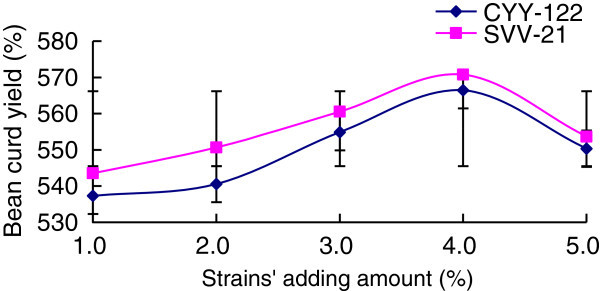
**Effects of strains’ adding amount on soymilk curd.**

Test each sample’s soybean curd yield and show the result on Figure 5, and then do the sensory evaluation.

From experimental results, for the strain CYY-122, before reaching the inoculation amount of 3.0%, soybean curd yield increased as strains’ adding amount increased, and it reached its top at the amount of 3.0%, but it dropped constantly from the amount of 3.0% to 5.0%. But for the strain SVV-21, its soybean curd yield reached the top at the amount of 4.0%. It is because with the increase in the amount of lactic acid bacteria inoculation, the rate of producing lactic acid of fermentation substrate rose and the amount of lactic acid increased, that made protein fully condense into gel state and as a result, the soybean curd yield increased. However, the soybean curd yield dropped constantly when inoculation the amount was beyond 4.0%. It is probably because too much lactic acid bacteria led to the competition for fermentation substrate between them, and thus affect their fermentation activity. In sensory evaluation of two strains, the block type was relatively complete, and it had a sense of particles as well as lack of fine texture, which meant soybean curd’s quality has not yet met the best in single strain fermentation. Accordingly, we should consider studying the influence on soybean curd’s quality from mixing two strains in fermentation.

### Effects of strains’ mixing ratio on soybean curd

Select the soy milk whose soluble solids content is 9.5% to inoculate CYY-12 and SVV-21 as the ratio of 1:1,1:2,2:1, respectively, and culture them under the temperature of 37°C for 5h. Measure each sample’s soybean curd yield and do sensory evaluation.

**Figure 6:**
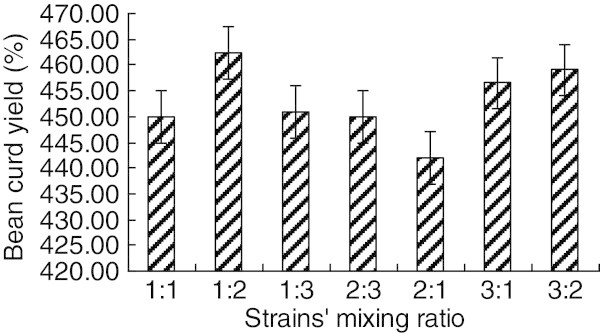
**Effects of strains’ mixing ratio on soymilk curd.**

Inoculate CYY-12 and SVV-21 as the ratio of 1:1,1:2,2:1, respectively, and measure each sample’s soybean curd yield, then show it on Figure 6.

From experimental results, the soybean curd yield reached its top at the ratio of 1:2 between CYY-122 and SVV-21, and the sample under this ratio got the highest grade in color, flavor, morphology and taste of sensory evaluation, those advantages were more obvious than other ratio. That is to say, two mixing strains can live together and ferment normally, and the fermentation result they got is better than single strain did. Hence, the best ratio of two strains (CYY-122 and SVV-21) is 1:2.

### Effects of thickener added amount on soybean curd

#### Effects of soluble starch added amount on the quality of soybean curd

Select the cooked soy milk whose soluble solids content is 9.5% and keep other condition unchanged, then add soluble starch as the proportion of 1.0%, 1.2%, 1.4%, 1.5% respectively. Inoculate and culture them under the temperature of 37°C for 5h. Measure each sample’s soybean curd yield and do sensory evaluation.

### Effects of xanthan gum added amount on the quality of soybean curd

Select the cooked soy milk whose soluble solids content is 9.5% and keep other condition unchanged, then add xanthan gum as the proportion of 0.5%, 0.7%, 0.9%, 1.0% respectively. Inoculate and culture them under the temperature of 37°C for 5h. Measure each sample’s soybean curd yield and do sensory evaluation.

### Effects of sodium alginate added amount on the quality of soybean curd

Select the cooked soy milk whose soluble solids content is 9.5% and keep other condition unchanged, then add sodium alginate as the proportion of 1.0%, 1.2%, 1.4%, 1.5% respectively. Inoculate and culture them under the temperature of 37°C for 5h. Measure each sample’s soybean curd yield and do sensory evaluation.

### Effects of carrageenan added amount on the quality of soybean curd

Select the cooked soy milk whose soluble solids content is 9.5% and keep other condition unchanged, then add carrageenan as the proportion of 1.0%, 1.2%, 1.4%, 1.5% respectively. Inoculate and culture them under the temperature of 37°C for 5h. Measure each sample’s soybean curd yield and do sensory evaluation.

**Figure 7:**
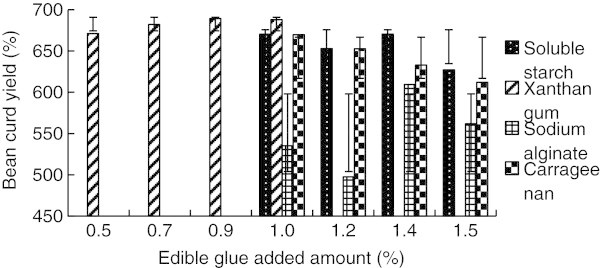
**Effect of edible glue adding amount on soymilk yield.**

The experimental results for the effect of edible glue adding amount on soybean curd yield was shown on Figure 7.

We can learn from the results above that adding soluble starch can increase soybean curd sharply, and makes the soybean curd block type more complete with soft and fine taste. But the aroma of bean is weaker with poor elasticity. Adding xanthan gum can as well increase soybean curd sharply, but the color of soybean curd appears more yellow and some colloids appear too. Also, the aroma of bean is weaker with poor elasticity. After adding sodium alginate, the increase of soybean curd is minor, but the aroma of bean is weaker and some colloids appear, as well as the elasticity is not enough, those are bad for the production control. Adding carrageenan can also increase soybean curd sharply, and makes the soybean curd block type more complete with soft and fine taste, but the aroma of bean is weaker and the elasticity is just general. Therefore, consider to optimize the crafts by complexing the edible glue. Soluble starch and carrageenan can be taken account to complex, and they both are not easily to produce colloids, which is in favor of the production control.

From the analysis of experimental results, the optimum conditions for the lactic acid bacteria to ferment curd is: Incubation temperature is 37°C, soy milk concentration (In terms of the content of soluble solids) is 12.5%, the ratio of CYY-122 and SVV-21 of lactic acid bacteria *Bulgarians* and *Streptococcus thermophilus* (CYY-122:SVV-21) in mixed fermentation is 1:2, the inoculation amount is 4.0% of soy milk volume, carrageenan adding amount is 1.4%, and incubation time is 5h.

### Orthogonal experiments of optimizing soybean curd processing conditions in lactic acid bacteria fermentation

**Figure 8:**
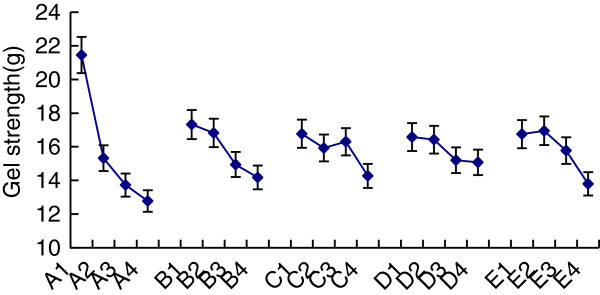
**Effect of various factors on gel strength of soymilk.**

According to the single factor experiment, we chose soy milk concentration (in soluble solids content), strains amount, edible glue amount, incubation time and temperature as the main factors in orthogonal experiments to find out the best conditions for lactic acid bacteria to ferment soybean curd. The observing indicators are gel strength and water holding capacity of soybean curd. Orthogonal factors have been shown on Table 3. Select L_16_ (4^5^) to do orthogonal experiment and have been shown on Table 4. Table 5 has shown the extreme difference analysis of gel strength as indicator, and Table 6 has shown the variance analysis of soybean curd gel strength. Figure 8 has shown the effect of various factors on gel strength of soybean curd.

**Table 3:** **Factors of L16 (45) orthogonal experiment**

	A	B	C	D	E
1	12.5	4.0	1.4	5.0	39
2	10.5	2.0	1.2	4.5	42
3	11.0	5.0	1.0	6.0	37
4	9.5	3.0	1.5	5.5	40

* A: soluble solids content/%, B: incubation amount/%, C: edible glue amount/%, D: time/h, E: temperature/°C.

**Table 4:** **Orthogonal experiment of soybean curd curding conditions**

A	A	B	C	D	E	F	G
1	12.5	4.0	1.4	5	39	25.6	69.82
2	12.5	2.0	1.2	4.5	42	24.3	59.67
3	12.5	5.0	1.0	6	37	20.4	63.05
4	12.5	3.0	1.5	5.5	40	15.5	48.28
5	10.5	4.0	1.2	6	40	14.3	45.08
6	10.5	2.0	1.4	5.5	37	16.5	44.09
7	10.5	5.0	1.5	5	42	14.8	43.90
8	10.5	3.0	1.0	4.5	39	15.7	30.48
9	11.0	4.0	1.0	5.5	42	16.1	40.16
10	11.0	2.0	1.5	6	39	13.5	55.16
11	11.0	5.0	1.4	4.5	40	12.4	52.03
12	11.0	3.0	1.2	5	37	12.9	46.77
13	9.5	4.0	1.5	4.5	37	13.3	43.44
14	9.5	2.0	1.0	5	40	13.0	34.00
15	9.5	5.0	1.2	5.5	39	12.2	30.85
16	9.5	3.0	1.4	6	42	12.6	42.83

*A: soluble solids content/%, B: incubation amount/%, C: edible glue amount/%, D: time/h, E: temperature/°C, F: gel strength/g, G: water holding capacity/%

**Table 5:** **Extreme difference analysis of gel strength**

G	A	B	C	D	E
1	21.450	17.325	16.775	16.575	16.750
2	15.325	16.825	15.925	16.425	16.950
3	13.725	14.950	16.300	15.200	15.775
4	12.775	14.175	14.275	15.075	13.800
Extreme difference	8.675	3.150	2.500	1.500	3.150
Better	A_1_	B_1_	C_1_	D_1_	E_2_

* A: soluble solids content/%, B: incubation amount/%, C: edible glue amount/%, D: time/h, E: temperature/°C.

**Table 6:** **Variance analysis of gel strength**

Sources of variance	Sum of squared deviations	F	Ratio of F	Critical value of F	Distinct-iveness
Soluble solids content	182.412	3	24.315	9.280	*
Incubation amount	26.952	3	3.593	9.280	
Edible glue amount	14.162	3	1.888	9.280	
Time	7.502	3	1.000	9.280	
Temperature	24.897	3	3.319	9.280	
Errors	7.50	3			

From experimental results, from the perspective of the gel strength, order of the impact of various factors on the results is: soluble solids content > incubation amount > temperature > edible glue amount > time. Under the confidence of 95%, only soluble solids content can be the distinctive factor, which is said that soluble solids content should be strictly controlled in soybean curd gel formation process. As a result, based on the results of the gel strength, the best combination of five factors is: A_1_B_1_C_1_D_1_E_2,_ that is, 12.5% of soluble solids content, 4.0% of incubation amount, 1.4% of edible glue amount, 5 hours of culture time and the temperature is 42°C.

Compare to the traditional fermentation methods, being coagulated by strains is not inconvenient than traditional soybean curd making methods and it can completely achieve the desired objectives. In addition, our products’ appearance is similar with the current products sold on markets, and there is no whey separation, which means, the methods used in this study is much better than traditional methods.

## Conclusions

Using the way of single factor in combination with orthogonal experiment, the process conditions of the lactic acid bacteria in soybean curd fermentation process was optimized. Parameters obtained from optimization are as follow: 12.5% of soluble solids content, 4.0% of incubation amount, 1.4% of edible glue amount, 5 hours of culture time and the temperature is 42°C. Under these conditions, the experimental result shows that, gel intensity has reached 25.6g, and water retention rate has reached 69.82%, which indicated that the optimized conditions are the best conditions of curd process.

Although we spend more time to coagulate than the traditional methods, our process is totally different with the traditional one which means there is no comparability between them. In our process, we emphasize the nutrition value rather than just economic profit. Furthermore, our process can move out the beany odor and provide products’ with cute flavor; also waste water is sharply decreased so that the process is environmental friendly.

## Methods

Raw soybean → Selection → Washing → Soak → Refining → Colloid mill → Filtering → Boiling pulp → Decreasing temperature → Adding thickener → Curd (inoculating lactic acid bacteria) → Keeping the temperature → Forming → Soybean curd

### Measurement of pH

The pH of curd was measured with a FE-20 pH meter at 15°C. Titratable acidity was quantified by titration to 10 mL of sample with 0.1 mol equi/L NaOH and expressed as concentration of lactic acid in g/100 mL. The pH meter is from Mettler - Toledo equipment Co., Ltd.

### Determination of yield of bean curd

Soybean curd was calculated as the weight (g) of fresh soybean curd obtained from 100g dry soybean used in percentile.

### Sensory evaluation

Color, flavor, morphology and texture are the critical sensory index that can determine the quality of curd, and it is a comprehensive reflection of curd’s sensory quality. Therefore, select the color, flavor, morphology and texture in this experiment as evaluated object in different culture time. A five-point scoring method has been used.

A sensory panel of 5 team members, who are majoring in food, was organized and do the sensory evaluation for lactic acid bacteria fermented soybean curd samples according to the following steps, respectively. Repeat those steps for 3 times with different orders for the whole evaluation, then calculate the average of all points. The full score of each index is 5.

i. The color of the samples is observed by the naked eye.
ii. Feel the morphology of the samples through the sense of touch (rub by thumb and index finger).
iii. Feel flavor characteristics of the samples through the sense of smell (with fingers to rub and then smell its taste).
iv. Feel the overall taste among the tongue, nose, teeth and mouth of the samples by chewing.

Scoring standards and details are as shown in the Table 7.

**Table 7:** **Form for sensory evaluation of products**

Index	Features	Deduction	Scores
Morphology	Neat block type, no broken edges, no missing angle		5
	Irregular block type	0.1 ~ 1.0	
	Broken edges	0.1 ~ 1.5	
	Loose	0.1 ~ 2.5	
Color	Oyster white		5
	Slight yellow	0.1 ~ 1.0	
	Lack of luster	0.1 ~ 2.0	
	Nonuniform color, and the color is not correct	0.1 ~ 2.0	
Taste	Delicate taste, flexible, good toughness, moderate hardness		5
	Delicate taste, but lack of flexible	0.1 ~ 1.0	
	The taste is a little rough, a little softer or stronger	0.1 ~ 2.0	
	Rough texture, obvious grainy feeling	0.1 ~ 2.0	
Flavor	Pure and accurate aroma, has the particular flavor of soybean curd gel, and no beany odor		5
	Aroma is not obvious , or lack of specific flavor of soybean curd gel	0.1 ~ 1.0	
	Inaccurate aroma and has slightly beany odor that affects the aroma	0.1 ~ 1.5	
	Bad smell , or full of beany odor	0.1 ~ 2.5	

As to the environmental sensory evaluation, since our process was finished in the laboratory, there was no waste water and other environmental pollution, so there is no need to do the environmental sensory evaluation. Thus, we did not conduct the environmental sensory evaluation.

### Making method of bean curd

#### Strains selection

In this study, we choose CYY-122 lactic acid bacteria *Bulgarians* and *Streptococcus thermophilus*, and SVV-21 lactic acid bacteria *Bulgarians* and *Streptococcus thermophilus* to finish the coagulation process. That is because, on the one hand, referring to yogurt production process, we use acid to coagulate and remove the beany flavor by fermentation, and strengthen the nutrition and health function of our product. In the early research of the study we have compared the fermentation effect of different strains, and it turned out that the strains we chose is the best. On the other hand, the strains we procured from Netherlands DSM Company are the commercial strains in current Chinese market. They are convenient and have stable source and do not need to subculture.

### Procedure

Our making method of bean curd has been shown in the very beginning of this chapter, and the reason why we did not choose to use traditional soybean curd making methods is because we used acid but not calcium to coagulate as traditionally did, and we focused more on our products’ nutrition function which is the different key selling point comparing to the traditional one. Besides, since acid coagulation and calcium coagulation products are not comparable, we did not compare the two methods in this paper.

### Statistical methods

Each of the data was tested for three times and then we calculated the average based on them. Thus those data shown on this paper is the average form.
